# Editorial: Functional Nanomaterials for Cancer Diagnostics and Therapy

**DOI:** 10.3389/fchem.2021.670410

**Published:** 2021-03-24

**Authors:** Wansong Chen, Jianhua Zhang, Xiaoli Wei

**Affiliations:** ^1^College of Chemistry and Chemical Engineering, Central South University, Changsha, China; ^2^Department of Polymer Science and Engineering, School of Chemical Engineering and Technology, Tianjin University, Tianjin, China; ^3^Department of Pharmacology, School of Basic Medical Sciences, Fudan University, Shanghai, China

**Keywords:** nanomaterials, diagnostics, drug delivery, bioimaging, cancer therapy

Efficient cancer therapy has been the research focus in biomedicine field for decades. Available therapeutic modalities for cancer treatment in clinic include surgery, chemotherapy, and radiotherapy. Surgery is difficult to completely remove tumors from normal tissues, and residual tumor cells are readily to relapse and result in treatment failure. In this regard, chemotherapy and radiotherapy are usually required to eradicate residual tumor cells after tumor resection by surgery. However, both chemotherapy and radiotherapy are challenged by serious side effects because of their off-target damage to normal tissues. Moreover, tumor cells are likely to activate anti-apoptotic signaling pathways to resist treatment. Very recently, immunotherapy has been demonstrated an promising way in fabricating long-term antitumor immunity to fight against cancer recurrence and metastasis, but only present efficacy to a subset of patients. In light of the above mentioned issues, it is imperative to develop more efficient and safe strategies for cancer treatment.

The interdisciplinary research of material chemistry and biomedicine provides more opportunities to overcome these challenges. Enormous endeavors have been devoted in the past decades from the following aspects.

(1) To improve the efficacy of chemotherapy or radiotherapy, intelligent drug nanocarriers have been designed and constructed to increase drug retention in tumors. Drugs are released from nanocarriers in response to specific tumor microenvironment, such as low pH or high glutathione level. Moreover, external stimuli (i.e., light, ultrasound, or magnetic field) have also been employed for drug controlled release.

(2) Phototherapeutic platforms have been developed based on some semiconducting materials or photosensitive agents, which can generate local hyperthermia or reactive oxygen species (ROS) under light irradiation. Since visible light hardly penetrates human tissues, near infrared (NIR) light with much deeper tissue penetrating depth has been widely investigated for phototherapy in recent years. In addition to light, ultrasound with extraordinary tumor penetrability has also been employed for generating ROS in tumors, which is termed as sonodynamic therapy.

(3) Tumor therapy drugs have been combined with imaging agents, such as upconversion nanoparticles, quantum dots, or fluorescence dyes. Under the guidance of imaging agents, both tumor location and drug delivery process can be facilely monitored, providing possibilities to achieve personal medicine and tumor theranostics.

(4) Besides, some nanomaterials themselves can be designed with therapeutic functions, such as radioprotection, anti-inflammation, or immune activation for tumor vaccines.

In this topic, we present original research and review articles with focus on functional nanomaterials for cancer diagnostics and therapy. In the direction of functional nanocarriers, Wang et al. reported a covalent organic framework nanomaterials as carriers for DOX loading, showing high drug loading capacity and pH-/redox-sensitive release in tumor cells. Howaili et al. synthesized a plasmonic nanogel composed of Au nanoparticles and polymer-based hydrogel nanoparticles. Such plasmonic nanogel was employed as drug carrier for curcumin delivery and dual pH-/photo-responsive release. In addition, Zhao et al. investigated the potential of black phosphorus quantum dots as carriers of Chinese herbal medicine for lymphoma therapy. Lin et al. synthesized a new kind of polymer nanocarriers with minimal cytotoxicity and high stability. The polymer nanocarriers improved antioxidant activity of curcumin, showing great potential as protection agent in caner radiotherapy. Gupta et al. developed solid lipid nanoparticles as carriers to improve the solubility, stability, and bioavailability of curcumin.

In the direction of phototherapy/sonodynamic therapy, Feng et al. developed a multifunctional nanosystem with bovine serum albumin as “mother ships” material. The nanosystem was demonstrated to be selectively accumulated within tumors, disrupt extracellular matrix for tumor penetration, and generate ROS to induce tumor cell apoptosis. This work offer a new avenue for improving photodynamic therapy via reprogramming tumor microenvironment. To improve tumor hypoxic environment, Huang et al. designed an oxygen-sufficient nanoplatform with polymer-perfluorocarbon nanoparticles as oxygen carriers. The nanoplatform not only improved the ROS generating efficiency of sonodynamic process, but also overcame tumor drug resistance to enhance therapeutic outcomes. As a Food and Drug Administration (FDA)-approved NIR photosensitizer, indocyanine green (ICG) has been widely used in oncology. Sevieri et al. summarized the applications and perspectives of ICG nanoparticles, offering insights for its further biomedical applications. Zhang et al. reviewed the progress of photodynamic therapy with internal light sources from the aspects of chemiluminescence, bioluminescence, and Cerenkov radiation, all of which circumvent the limitation of light penetration in body. Jia et al. coated Fe_3_O_4_ with mesoporous silica and polydopamine for drug delivery and synergistic photothermal/chemodynamic therapy.

In the direction of cancer diagnostics and theranostics, Yu et al. reviewed the recent progress of rare-earth-doped nanoparticles for tumor fluorescence imaging and theranostics in the second near infrared window (NIR-II, 1,000–1,700 nm). Hong et al. reported theranostic platform based on upconversion nanoparticles, which can accumulated in the mitochondria of tumor cells. Under NIR light irradiation, such nanoplatform emitted fluorescence signal for imaging-guidance and produced ROS for tumor killing. Tang et al. reviewed the advances of noble metal nanoclusters (Au and Ag) in biosensing, bioimaging, and cancer therapy.

Besides, Shang et al. studied antitumor activity and tumor-killing mechanism of zirconia nanoparticles (ZrO_2_), providing a promising alternative to traditional chemotherapy drugs for tumor treatment. When camouflaged with platelet membrane (PLT), the obtained PLT@ZrO_2_ nanoparticles successfully escaped from immune recognition and targeted tumor cells, thereby achieving prolonged blood circulation time and tumor targeting delivery Shang et al.. Seré et al. reported a nanovaccine based on ovalbumin-conjugated mesoporous silica nanoparticles, which was attractive as a replacement of dendritic cell-based vaccines.

We hope this Research Topic will provide researchers information to understand the advanced strategies of functional nanoplatforms in cancer diagnostics and therapy, inspiring new ideas for future research directions and research activities.

## Author Contributions

All authors listed have made a substantial, direct and intellectual contribution to the work, and approved it for publication.

## Conflict of Interest

The authors declare that the research was conducted in the absence of any commercial or financial relationships that could be construed as a potential conflict of interest.

